# Rituximab in idiopathic nephrotic syndrome: still waiting for stronger evidences

**DOI:** 10.1590/2175-8239-JBN-2023-E012en

**Published:** 2023-10-06

**Authors:** Vera Maria Santoro Belangero

**Affiliations:** 1 Universidade Estadual de Campinas, Faculdade de Ciências Médicas, Departamento de Pediatria: Nefrologia Pediátrica, Campinas, SP, Brazil.

Gomes et al.^1^ present in the current issue of BJN a report on the promising outcomes achieved with the use of rituximab (RTX) in 16 pediatric and adolescent nephrotic patients from Portugal. Their findings demonstrate a reduction in the frequency of relapses, a decrease in steroid dosage, and improvements in the patients’ bone mass index after RTX, which is consistent with existing literature.

Since the publication of the 2004 report on remission of nephrotic syndrome (NS) using RTX for thrombocytopenic purpura^2^, the literature on RTX in NS has expanded significantly. The journey from incidental use to evidence-based practice has been long and hard.

In the context of RTX in idiopathic nephrotic syndrome (INS), two factors pose challenges and hinder the establishment of robust evidence-based recommendations. First, there is a lack of more specific stratification of NS. In the era of molecular biology and remarkable discoveries in NS genetics, it is becoming increasingly evident that NS, including steroid-sensitive nephrotic syndrome (SSNS), encompasses a spectrum of diseases with multiple phenotypes^3^. Recent genome-wide association studies have identified various HLA and non-HLA risk loci involved in adaptive immunity in childhood-onset SSNS^4^. Therefore, traditional clinical characterization based on the initial response to corticosteroid therapy, number of relapses over time, or steroid dependence may not adequately delineate homogeneous groups in terms of disease pathogenesis and, consequently, therapeutic responses. Furthermore, the relatively recent use of RTX as a treatment option has led to substantial variations in treatment schedules, including differences in dosage, number of infusions, maintenance medications following RTX, duration of follow-up, and criteria for selecting cases^5^.

Despite the complexity of the literature, Chan et al.^5^ conducted an extensive review that identified factors associated with the efficacy of RTX treatment in INS. The review found that improved treatment outcomes were associated with older age at the time of RTX administration, white ethnicity in children, utilization of maintenance medications following RTX, repeated courses of RTX, and favorable baseline immunologic profiles (including a higher number of circulating regulatory T-cells and lower mitogen-stimulated T-cell subsets)^5^. Conversely, steroid resistance, dependence on multiple medications, low RTX dosage without concurrent maintenance medication, and rapid repopulation of total memory B cells were associated with a higher risk of relapse. It has been observed that steroid sensitivity (SS) prior to the administration of RTX is strongly correlated with favorable effects of RTX treatment, not only in young children and adolescents but also in adults^6^. Furthermore, a longer remission was noted in patients who had exclusively used prednisone prior to receiving RTX^7^. In a large retrospective study, it was found that each additional steroid-sparing agent (SSA) used prior to RTX was associated with a 19% increase in the risk of relapse following RTX treatment^8^. These findings highlight the importance of considering the patients’ prior steroid responsiveness and number of previous SSA use as potential factors influencing treatment outcomes with RTX.

In 2023, the International Pediatric Nephrology Association (IPNA) published recommendations regarding the treatment of INS, which included the use of RTX. These recommendations were developed based on the RIGHT (Reporting Items for Practice Guidelines in Healthcare) Statements for Practice Guidelines. The development of the guideline involved three groups: a core leadership group, an external expert group, and a voting panel of 32 pediatric nephrologists with expertise in managing INS^7^. This approach is particularly suitable when there is considerable methodological variation in the available literature methodology and expert opinion can provide valuable insight into the field.

The IPNA recommendations comprehensively cover all relevant aspects of RTX use in INS, including indications, dosage, treatment regimen, and use of maintenance medications following RTX. According to these recommendations, RTX is indicated as a second-line option to be considered after a course of treatment with at least one other SSA. This differs from the stance of some authors who advocate for RTX as a first-line option, particularly in patients with milder disease who may potentially respond to steroids or other better-established SSA options^5^.

By emphasizing the need for prior treatment with SSA before considering RTX, the IPNA recommendations provide a more conservative approach to RTX utilization in INS, taking into account the potential benefits and risks associated with this therapeutic option.

Most studies suggest that RTX is reasonably safe in the short term and relatively effective compared to other SSA, as also demonstrated by Gomes et al.^1^. However, unlike other immunosuppressive therapies, there is a lack of long-term follow-up data on RTX use in INS. To address this gap, the European Society of Pediatric Nephrology (ESPN) conducted a survey in 84 centers, involving 1328 children, to gather information on RTX-associated hypogammaglobulinemia and its potential consequences in INS^9^. This survey provides valuable insights as it reflects the common practice of pediatric nephrologists outside of research protocols and includes a robust number of patients. Although the frequency of hypogammaglobulinemia could not be calculated due to study design limitations, persistent hypogammaglobulinemia was observed in 30 out of 34 severe infections, and four patients died.

Epidemiological studies focusing on INS in recent decades have been limited. In a Canadian cohort of 631 patients followed for a median of 3.9 years, no deaths were reported^10^. In a nationwide study conducted in Japan, including 2099 children with INS followed for 1 to 4 years, two deaths occurred during the study period, with only one death associated with INS^11^. Therefore, the guideline for the treatment of INS strongly advises against the use of high-risk therapies. However, for difficult-to-treat patients who have experienced toxicity from previous therapies, there is a need to explore more effective treatment options. Whether RTX can resolve this dilemma remains a topic of ongoing debate. Pediatric nephrologists have been facing challenges in guiding their patients and attempting to individualize treatment approaches based on available scientific evidence.

In the midst of this ongoing debate, the IPNA recommendations are timely. They carefully weigh the risks and benefits and provide a practical guide to current practice while laying the foundation for future scientific studies.
